# Supplementary material for: Superplasticity in an organic crystal
Source: Nat Commun. 2018 Sep 28;9:3984. doi: 10.1038/s41467-018-06431-7 (PMC6162311; doi:10.1038/s41467-018-06431-7)
Supplement: Supplementary file 1 — Supplementary Information [file 41467_2018_6431_MOESM1_ESM.pdf]

Supplementary information: Superplasticity in an organic crystal

Takamizawa et al.

Superplasticity, which enables processing on hard-to-work solids, has been recognized only in metallic solids. While metallic materials and plastics (polymer solids) essentially possess high plastic workability, functional crystalline solids present difficulties in molding. Organic crystals especially are fragile, in the common view, and they are far from the stage of materials development. From the viewpoint of practical application, however, organic crystals are especially attractive because they are composed of ubiquitous elements and often exhibit higher performance than metallic materials. Thus, finding superplastic deformation of organic crystals, especially in a single-crystal-to-single-crystal manner, will pave the way to their material applications. This study confirmed superplasticity in a crystal of a simple organic compound: *N*,*N*-dimethyl-4-nitroaniline. The crystal exhibits single-crystal-to-single-crystal superplastic deformation without heating. This finding of "organosuperplasticity" will contribute to the future design of functional solids that do not lose their crystalline quality in molding.

Contents: **Page**

(a) Supplementary Note 1. Exprimental information **2**

(b) Supplementary Note 2. Variations of plastic deformation **3-4**

(c) Supplementary Note 3. Detailed information of crystal structure **5-10**

(d) Supplementary Note 4. Information and data on the observations of superplasticity **11-13**

Supplementary references **14**

Other materials:

Supplementary Movie 1: Superplastic deformation of a crystal of **1** up to ca. 500% strain.

Supplementary Movie 2: Twinning deformation of a crystal of **1** in conjunction with multi-layer slipping.

Supplementary Movie 3: Superelastic behavior of a crystal of **1**.

Supplementary Movie 4: Superplastic deformation of a crystal of **1** of which strain rate quickly shifted from 1.0 s^{-1} to 5.0 s^{-1}.

Supplementary Movie 5: Superplastic deformation of a crystal of **1** of which strain rate quickly shifted from 2.0 s^{-1} to 20 s^{-1}.

Supplementary Movie 6: Superelastic behavior of a crystal of **1** during a stress test.

Supplementary Movie 7: Superplastic deformation of a crystal of **1** up to ca. 400% strain.

Supplementary Movie 8: Superelastic behavior of a crystal of **1** after superplastic deformation up to ca. 400% strain.

Supplementary Movie 9: Coupling of superplasticity and superelasticity in a crystal of **1**.

(a) Supplementary Note 1. Exprimental information

Crystal preparation

N,*N*-Dimethyl-4-nitroaniline (**1**) was purchased from Tokyo Chemical Industry and acetone was purchased from Wako. Both were used as received. Well-formed single crystals were prepared by recrystallization of **1** from a concentrated solution in acetone.

Single crystal X-ray structural analysis

Single-crystal X-ray diffraction measurements of crystals of **1** were performed at 298 K (25 °C) on a Bruker SMART BREEZE CCD area diffractometer (Bruker AXS K.K.) with a nitrogen-flow temperature controller using graphite-monochromated MoKα radiation (λ = 0.71073 Å). Empirical or multi-scan absorption corrections were applied using the SADABS program. The structure was solved by intrinsic phasing methods (SHELXS-97 or SHELXT-2014/5) and refined by full-matrix least-squares calculations on F^2 (SHELXL-97 or SHELXL-2014/7). Non-hydrogen atoms were refined anisotropically; hydrogen atoms were fixed at calculated positions by riding model approximation. Crystallographic data of the structures are summarized in Supplementary Table 1. Crystal face indexing was carried out using APEX III Ver.2016.1-0 program package with a twin resolution program. CCDC 1847179-1847182 and 1858314 contain the supplementary crystallographic data.

Microscope observations

A polarization microscope coupled with a digital camera was used to record mechanical twinning using tweezers.

Force measurements

Stress tests were carried out on a universal testing machine (Tensilon RTG-1210, A&D Co. Ltd.). Conditions and results of shear tests are summarized in Supplementary Note 4 (Supplementary Table 2 and Supplementary Figures 9–11).

(b) Supplementary Note 2. Variations of plastic deformation

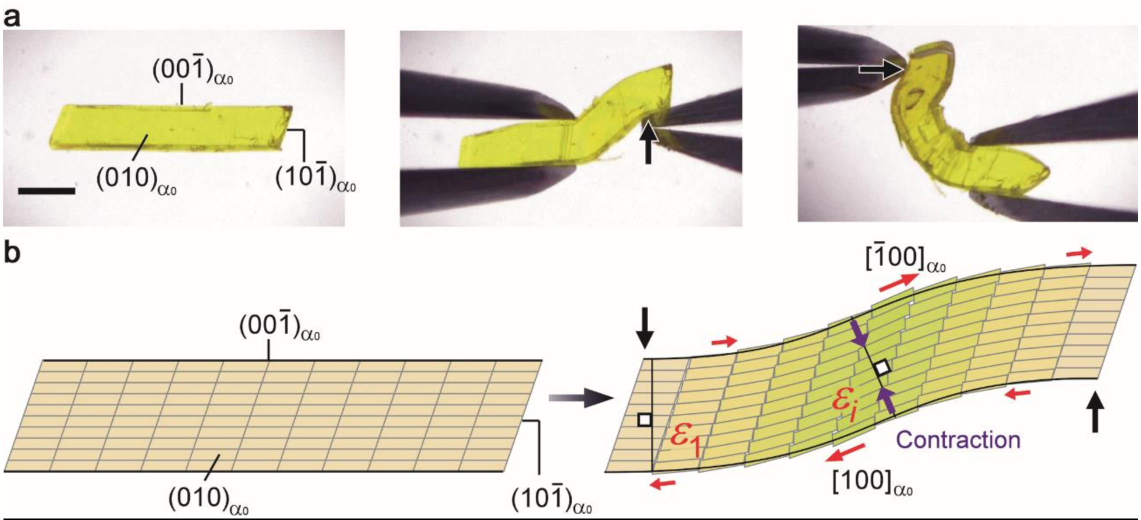

Supplementary Fig. 1. Plastic deformation of a crystal of **1**. **a** Plastic deformation by stress perpendicular to the {001} slip planes in superplasticity of a crystal of **1** at room temperature. Scale bar, 1.0 mm. **b** Schematic model explaining the S-shape crystal bending caused by interlayer shear along the <100> direction accompanied by contraction along the thickness direction of layers. The depth of color of the rectangles corresponds to the magnitude of shear strain. Inversion of the force direction (black arrows) in the above model results in inversion of the shear stress direction on each layer along the <100> direction. In such cases, beam bending was observed in single crystals of **1** accompanying twinning deformation as shown at the bottom of Figure 1c in the paper.

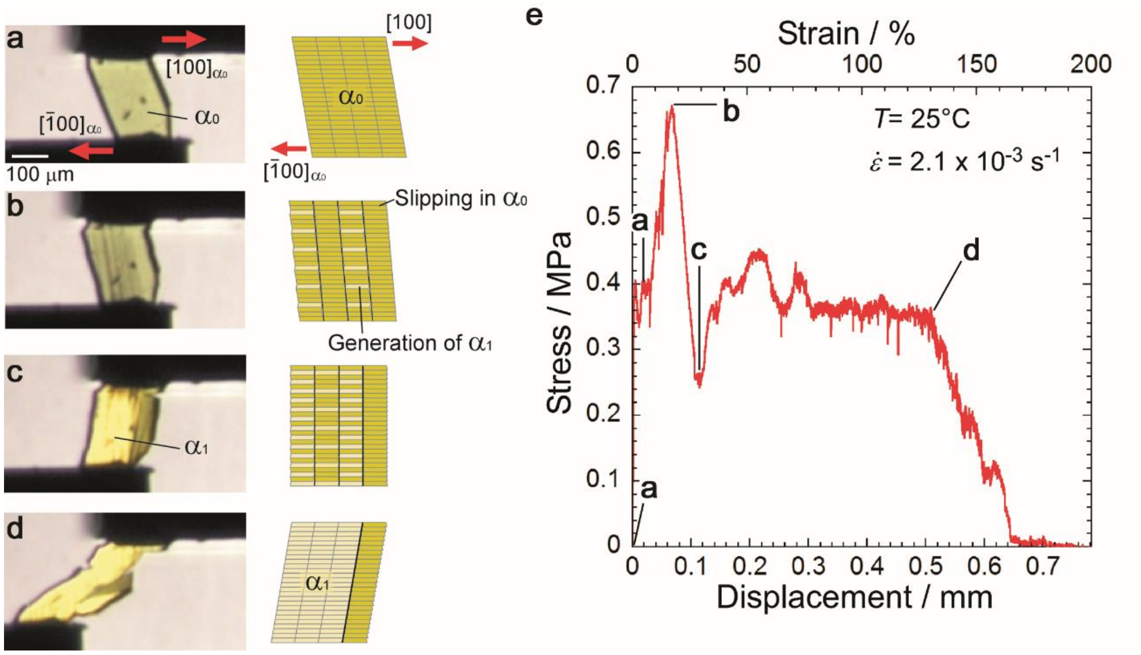

Supplementary Fig. 2. Shear test of a crystal of **1**. **a–d** Deformation behavior of a single crystal of **1** under shear stress in the direction opposite to the superplasticity-inducing vector at room temperature: snapshots (left) and schematic representation of the deformation (right). Scale bar, 100 μm. **e** Stress–strain curve of the crystal. The shear test began in the state where shear and molecular tilt were in opposite directions (**a**). Linear domains perpendicular to the shear direction were generated under the peak stress (**b**) in the stress-strain curve. Such domains increased in number as the strain increased (**b**–**d**). Shear stress in the slip deformation after the generation of the domains (0.35–0.4 MPa) agreed with the shear stress during the superplastic deformation in Figure 2 in the paper (0.30–0.35 MPa). This fact supports the observation that the crystal of **1** exhibited twinning deformation in conjunction with multi-layer slipping. (See Supplementary Movie 2.)

(c) Supplementary Note 3. Detailed information of crystal structure

Supplementary Tab. 1. Crystallographic data of **1** for as-prepared (original), superelastically twinned (mother and daughter domains), and superplastically deformed (the deformation rate: 100–150% and 330%) crystals.

Crystal	As-prepared crystal	Superelastically twinned crystal		Superplastically deformed crystal	
State	Original	Mother domain	Daughter domain	100–150% deformation	330% deformation
T /K	298	298	298	298	298
Empirical formula	$C_8H_{10}O_2N_2$	$C_8H_{10}O_2N_2$	$C_8H_{10}O_2N_2$	$C_8H_{10}O_2N_2$	$C_8H_{10}O_2N_2$
Crystal size /mm^3	0.45x0.15x0.05	0.60×0.25×0.12	0.60×0.25×0.12	0.55x0.17x0.06	0.12x0.19x1.05
M	332.36	332.36	332.36	332.36	332.36
Crystal system	Monoclinic	Monoclinic	Monoclinic	Monoclinic	Monoclinic
Space group	$P2_1$	$P2_1$	$P2_1$	$P2_1$	$P2_1$
a /Å	3.9705(7)	3.9630(10)	3.9573(13)	3.9720(18)	3.9816(16)
b /Å	10.5740(18)	10.564(3)	10.547(4)	10.579(5)	10.588(4)
c /Å	9.7323(16)	9.724(3)	9.710(3)	9.741(5)	9.752(4)
α /°	90	90	90	90	90
β /°	91.341(4)	91.278(6)	91.285(7)	91.316(5)	91.438(6)
γ /°	90	90	90	90	90
V /Å^3	408.49(12)	407.0(2)	405.2(2)	409.2(3)	411.0(3)
Z	2	2	2	2	2
D_{calcd} /Mg m^{-3}	1.351	1.356	1.362	1.349	1.343
μ(Mo Kα) / mm^{-1}	0.099	0.099	0.100	0.099	0.099
Reflections collected	3078	2307	2268	1844	2158
Independent reflections (R_{int})	1164 (0.0226)	1148 (0.0245)	1070 (0.0409)	823 (0.0273)	784 (0.0525))
Goodness of fit	1.070	1.181	1.218	1.037	0.946
$R_1(I > 2\sigma$ (all data))	0.0514 (0.0839)	0.0660 (0.0782)	0.0876 (0.1051)	0.0375 (0.0592)	0.0464 (0.0892)
$_wR_2(I > 2\sigma$ (all data))	0.1313 (0.1707)	0.1721 (0.1977)	0.2266 (0.2609)	0.0895 (0.0971)	0.1168 (0.1356)
Largest diff. peak (hole) /eÅ^3	0.230 (-0.262)	0.469 (-0.342)	0.589 (-0.413)	0.129 (-0.101)	0.154 (-0.190)
Hydrogen bonds* [-N(CH_3)$_2$···O_2N-]	3.610(6) (C8···O2$^{\#1}$)<br>3.588(6) (C7···O1$^{\#1}$)	3.600(7) (C8···O2$^{\#2}$)<br>3.600(7) (C7···O1$^{\#2}$)	3.589(9) (C8···O2$^{\#2}$)<br>3.595(9) (C7···O1$^{\#2}$)	3.621(6) (C8···O2$^{\#2}$)<br>3.605(6) (C7···O1$^{\#2}$)	3.621(6) (C8···O2$^{\#1}$)<br>3.605(6) (C7···O1$^{\#1}$)
CCDC No.	CCDC 1847179	CCDC 1847180	CCDC 1847181	CCDC 1847182	CCDC 1858314

*Symmetry operation: (x+1, y, z+1) $^{\#1}$ and (x-1, y, z-1) $^{\#2}$.

Retention of crystallinity

The crystals of **1** maintain their crystallinity during not only the superplastic deformation but also the superelastic twinning. As-prepared, superelastically twinned, and superplastically deformed crystals of 1 were examined by viewing the raw X-ray diffraction data (Supplementary Figures 3–6) converted to reciprocal space. The superelastically twinned crystals of 1 exhibited merged reflections of mother and daughter domains. (Supplementary Figure 4b(middle)). The superplastically deformed crystals of **1** exhibited ideal reflections, which were essentially identical to those of the as-prepared crystals (Supplementary Figures 5 and 6), while a certain tailing at some spots was eventually observed in the largely deformed crystals (deformation rate of 330%) through our experimental techniques. The observed manner of tailing of the spots on the reflections from the (h0l) plane in the raw diffraction data is most likely due to a slight deflection in the <001> direction of the stretched thin crystal specimen (Supplementary Figure 6b(middle)).

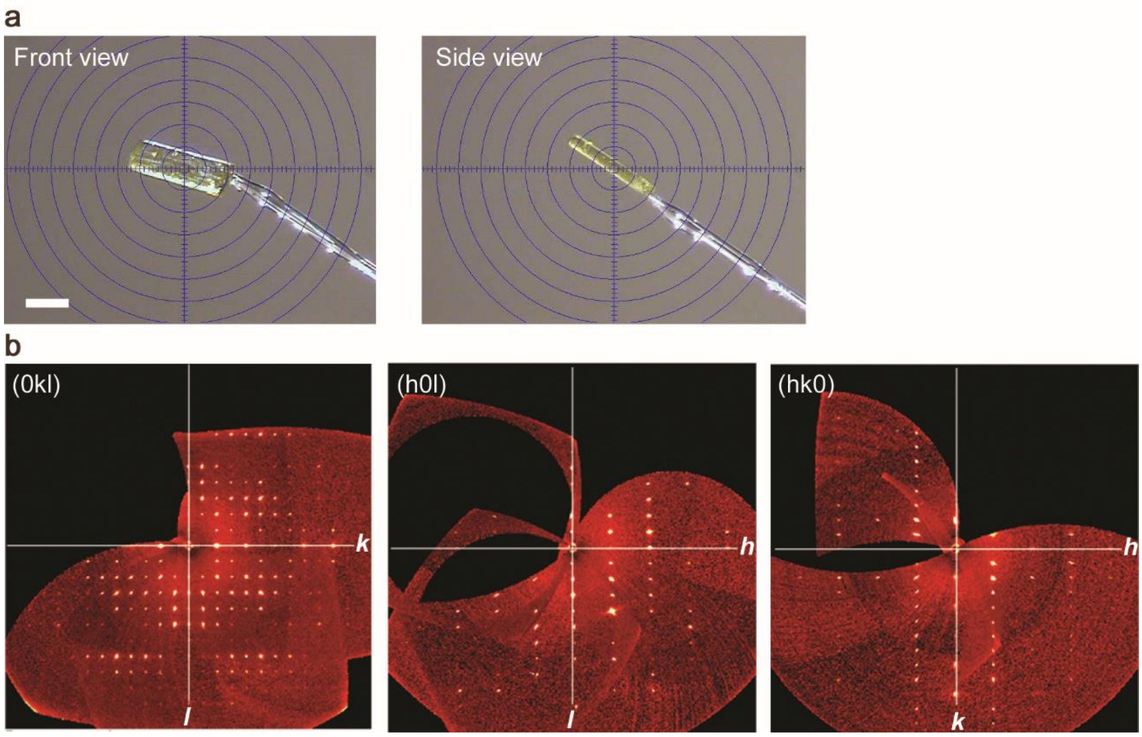

Supplementary Fig. 3. X-Ray diffraction measurements of a crystal of **1**: as-prepared. **a** Front view (left) and side view (right) of as-prepared (original) crystal of **1** used for single-crystal X-ray diffraction measurements (scale bar, 0.2 mm), **b** precession images (0kl) (left), (h0l) (middle), and (hk0) (right) planes calculated from the raw X-ray diffraction data. The obtained crystallographic data are summarized in Supplementary Table 1.

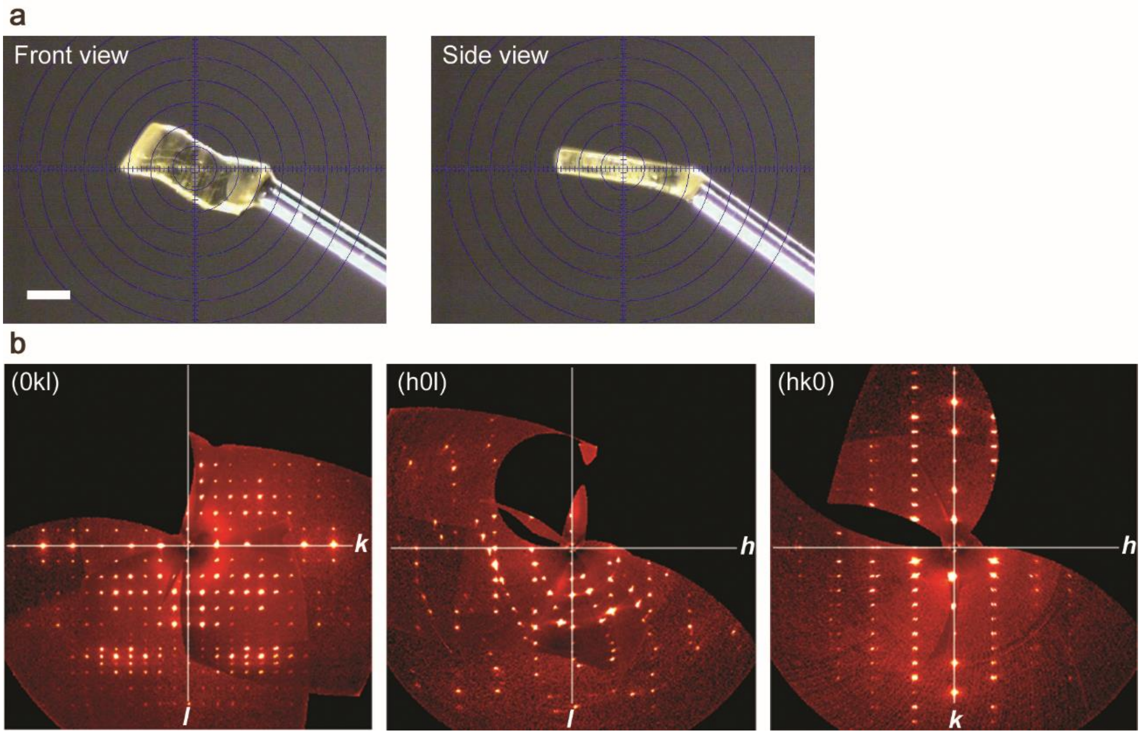

Supplementary Fig. 4. X-Ray diffraction measurements of a crystal of **1**: twinned. **a** Front view (left) and side view (right) of twinned crystal of **1** used for single-crystal X-ray diffraction measurements (scale bar, 0.2 mm), **b** precession images (0kl) (left), (h0l) (middle), and (hk0) (right) planes calculated from the raw X-ray diffraction data. The obtained crystallographic data are summarized in Supplementary Table 1.

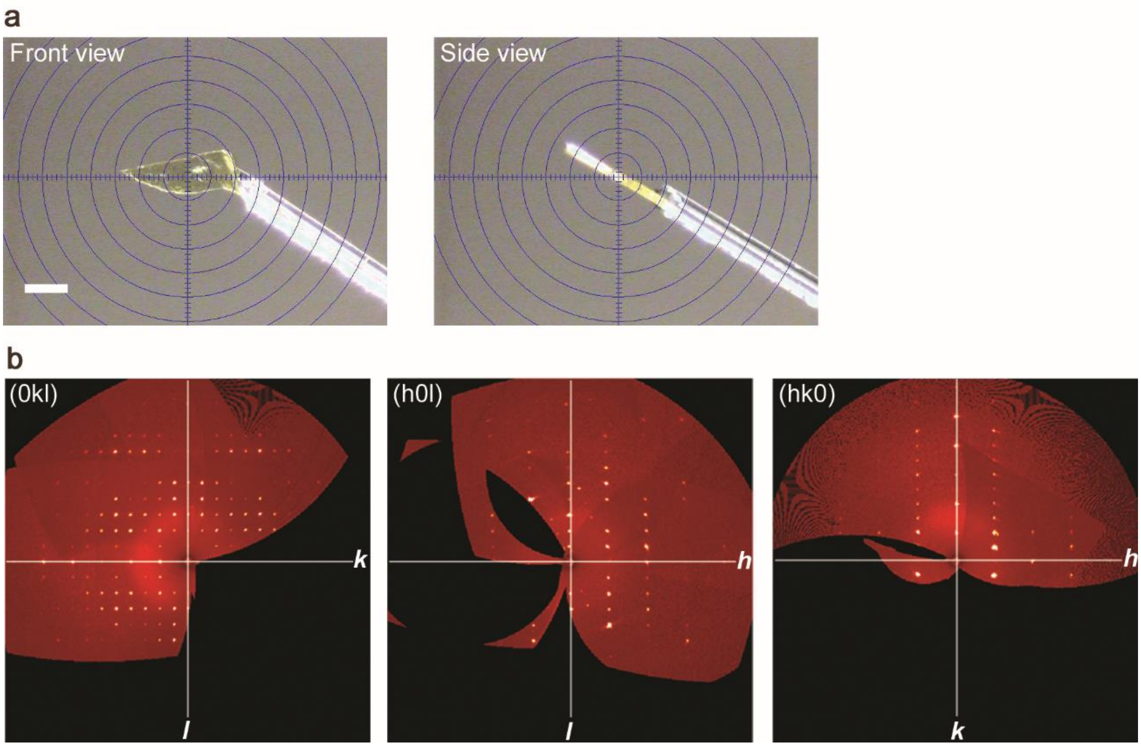

Supplementary Fig. 5. X-Ray diffraction measurements of a crystal of **1**: superpelastically deformed (100–150%). **a** Front view (left) and side view (right) of superplastically deformed crystal of **1** (the deformation rate: 100–150%) used for single-crystal X-ray diffraction measurements (scale bar, 0.2 mm), **b** precession images (0kl) (left), (h0l) (middle), and (hk0) (right) planes calculated from the raw X-ray diffraction data. The obtained crystallographic data are summarized in Supplementary Table 1.

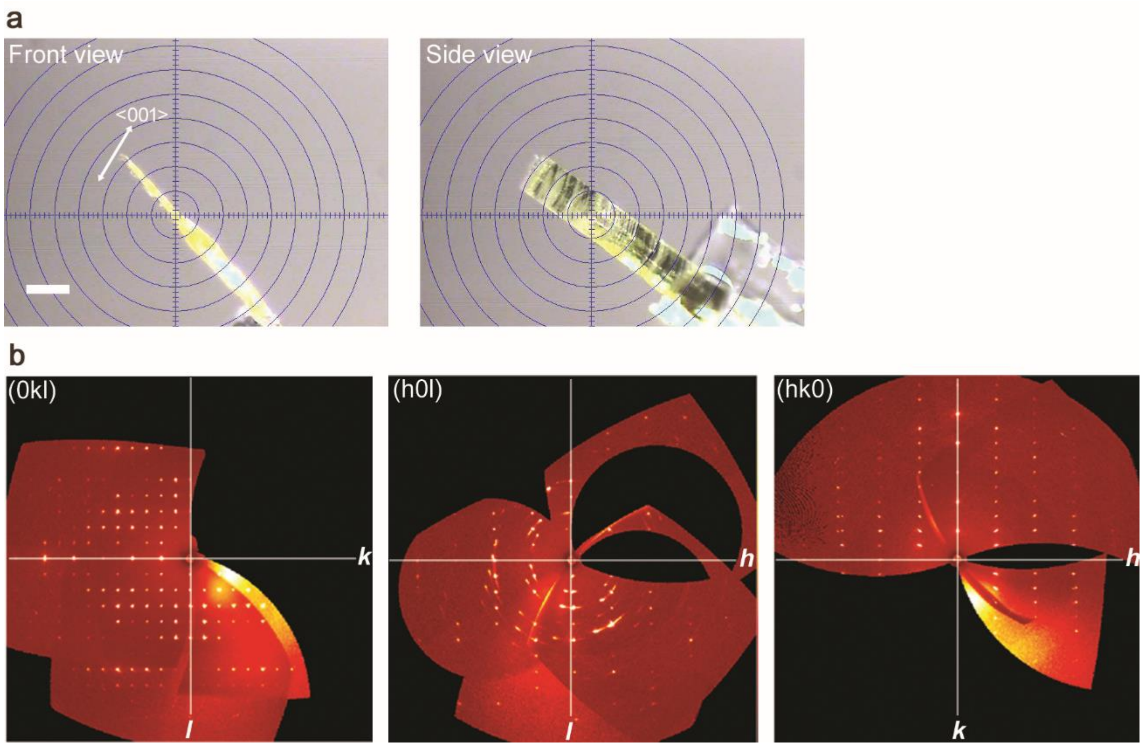

Supplementary Fig. 6. X-Ray diffraction measurements of a crystal of **1**: superpelastically deformed (330%). **a** Front view (left) and side view (right) of superplastically deformed crystal of **1** (the deformation rate: 330%) used for single-crystal X-ray diffraction measurements (scale bar, 0.2 mm), **b** precession images (0kl) (left), (h0l) (middle), and (hk0) (right) planes calculated from the raw X-ray diffraction data. The obtained crystallographic data are summarized in Supplementary Table 1. The tailing in the reflections from (h0l) plane is most likely due to the slight deflection in the <001> direction of the thin crystal specimen.

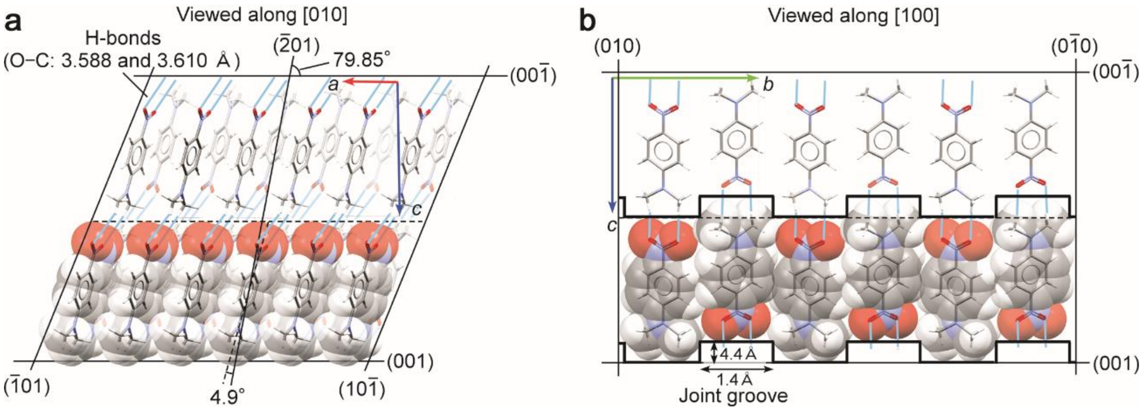

Supplementary Fig. 7. Packing diagram of the crystal structure of **1**, viewed parallel to a slip plane of superplastic deformation: **a** Viewed along the [100] direction and **b** Viewed along the [010] direction. The zigzag lines in **b** schematically represent grooves on slip planes considering the van der Waals radius of component molecules (width: ca. 4.4 Å, depth: ca. 1.4 Å). The blue dotted lines show [C-H···O = N] hydrogen bonds with the O···C distance of 3.588 and 3.610 Å.

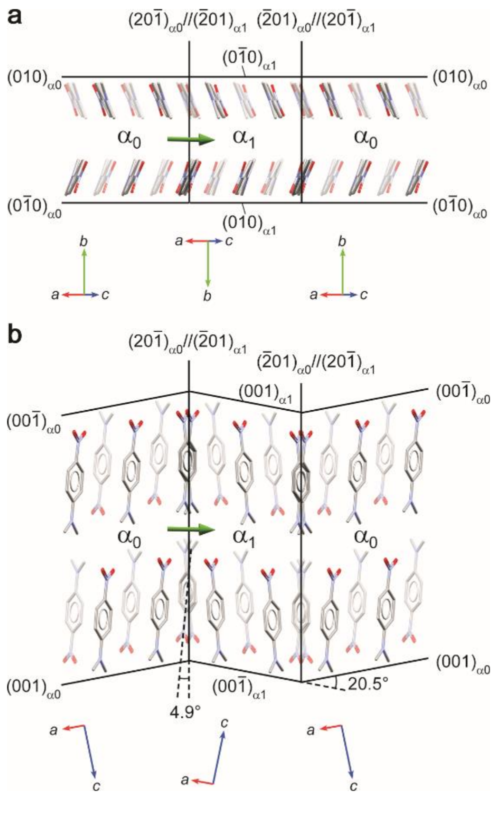

Supplementary Fig. 8. Packing diagram of a crystal of **1**, viewed parallel to interfaces in superelastic (pseudoelastic) deformation at room temperature: **a** Viewed along the shearing axis [102] and **b** Viewed down the *b* axis. The bold green arrows represent the rotational axis of the crystal lattice in twinning deformation.

(d) Supplementary Note 4. Information and data on the observations of superplasticity

Supplementary Tab. 2. Conditions of shear tests for observing slip deformation in crystals of **1**.

Temperature / °C	Shear direction	Slide plane	Crystal dimension		Velocity / µm min^{-1} (Strain rate / s^{-1})	Corresponding Figure
			Height / µm	Thickness / µm		
25	[-100]	{001}	338	55	50 (0.0025)	Figures 1 and 2 in the paper
25	[100][a]	{001}	391	58	50 (0.0021)	Supplementary Figure 2
25	[001][b]	-	331	28	50 (0.0025)	Figure 3 in the paper
25	[-100]	{001}	338	55	50 (0.0025)	Supplementary Figure10a
			300	36	1.8×10^4 (1.0)	Supplementary Figure 10b
					9.0×10^4 (5.0)	
			252	26	3.0×10^4 (2.0)	Supplementary Figure 10c
					3.0×10^5 (20)	

(a) Conducted by inverting the crystal orientation from that in the experiment in Figure 1 in the paper.
(b) No slip deformation.

Strain rate sensitivity index: *m*

Generally, an effective strain rate ($\dot{\varepsilon}_{eff} = v/h_{def}$) increases by decreases in a strain area ($h_{def} < h$) in slip deformation proceeding at a constant strain rate $\dot{\varepsilon}$ (= v/h). The relationship between deformation stress σ and effective strain rate $\dot{\varepsilon}_{eff}$ is represented empirically by $\sigma = K\dot{\varepsilon}_{eff}{}^{m}$ (K is a constant relating to solids).[S1] Superplasticity of metal and ceramic materials by grain boundary sliding has a high strain rate sensitivity index m (= $d(\ln\sigma)/d(\ln\dot{\varepsilon}_{eff})$), i.e. 0.3–1.0. Thus the critical slipping stress increases sharply when $\dot{\varepsilon}_{eff}$ increases. Consequently, localized solid deformation is suppressed, leading to easy slip deformation of whole solids in superplastic deformation according to previous reports.[S2,S3] From the viewpoint of practical use, however, superplastic deformation has a slow strain rate (around 10^{-4}–10^{-2} s^{-1}) and narrow range because of a high strain rate sensitivity index. Therefore, improvement of processing speed is important to plastic processing exploiting superplasticity.[S4,S5]

Estimation of *m* in crystals of **1**

The *m* values in superplastic deformation of crystals of **1** were investigated. The distance between the (001) and (00-1) faces (*h*) and the sliding rate of the plate (*v*) were 338 μm and 50 μm min^{-1} (0.833 μm s-^{1}), respectively. Thus the shear strain rate ($\dot{\varepsilon}$, *v*/*h*) was estimated as 2.5×10^{-3} s^{-1}. Using several crystalline samples, stress-strain curves were measured at room temperature by varying the strain rate, $\dot{\varepsilon}$, in the region of 2.5×10^{-3} – 20 s^{-1}. The detected stress increased from ca. 0.27 to 0.5 MPa when $\dot{\varepsilon}$ increased (Supplementary Figures 9 and 10). Accordingly, the *m* values of crystals of **1** were calculated as 0.0365–0.137 from the slopes on the graph in which the vertical and horizontal axes represent log$\dot{\varepsilon}$ and log σ, respectively. Such low values have not been confirmed in the superplasticity produced by grain boundary sliding of polycrystalline metals. This means that crystals of **1** can exhibit excellent superplastic deformation, which is difficult to find in superplasticity of metals, i.e. rapid superplastic deformation under almost constant deformation stress in a wide range of distortion rates (10^{-3}–10^{1} s^{-1}).

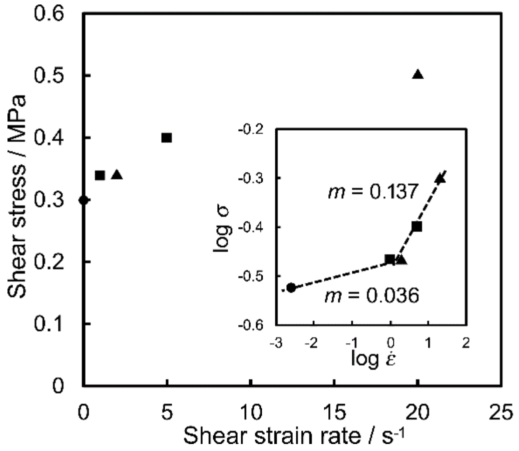

$\dot{\varepsilon}$ (s^{-1})	σ (MPa)
0.0025	0.30
1	0.34
2	0.34
5	0.40
20	0.50

Supplementary Fig. 9. Results of shear tests under quick strain rate shifts at room temperature. Left: Graph of the strain rate ($\dot{\varepsilon}$) dependence of deformation stress σ during superplastic deformation (solid circle: $\dot{\varepsilon}$ = 0.0025 s^{-1}, solid square: $\dot{\varepsilon}$ = 1.0 s^{-1} to 5.0 s^{-1}, solid triangle: $\dot{\varepsilon}$ = 2.0 s^{-1} to 20 s^{-1}). Right: Values of strain rates and deformation stress in the graph. The deformation stress values were averaged values during homogeneous deformation. The slopes in the inset of the graph represent strain rate sensitivity index *m*.

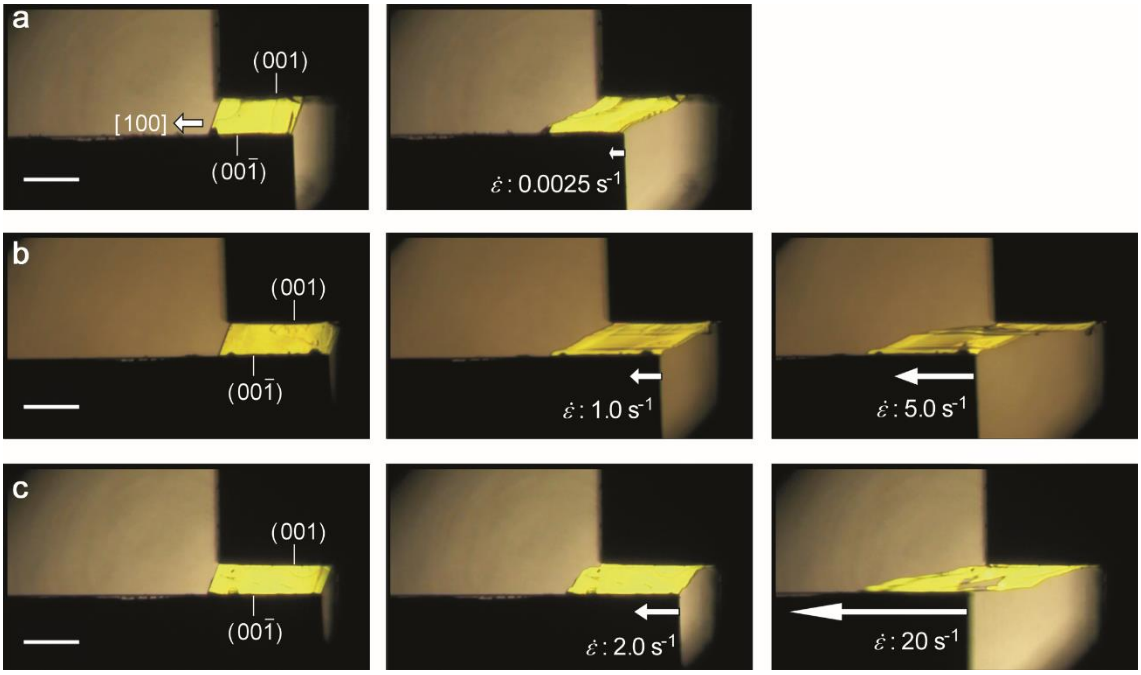

Supplementary Fig. 10. Snapshots from the shear tests in Supplementary Figure 9 under various strain rates. The shear direction and crystal orientation are the same as with the crystals in Figures 1 and 2 in the paper. **a** Snapshots during the experiment in Figure 2 in the paper (0.0025 s^{-1}). (Refer to Supplementary Movies 1.) Strain rates shifted quickly: **b** From 1.0 s^{-1} to 5.0 s^{-1} and **c** From 2.0 s^{-1} to 20 s^{-1}. (Refer to Supplementary Movies 4 and 5.) Scale bar, 500 μm.

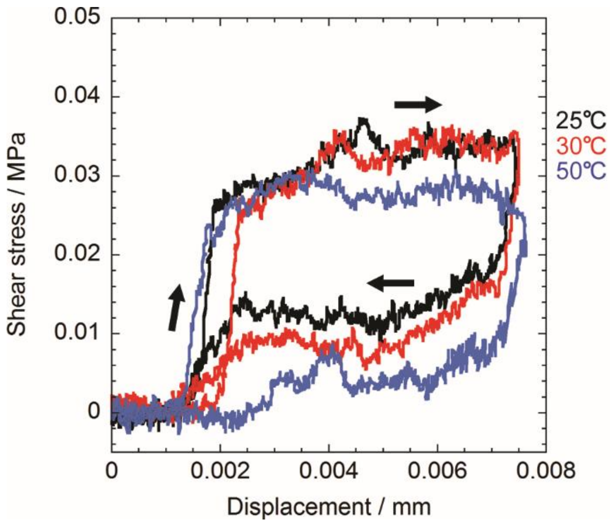

Supplementary Fig. 11. Stress-strain curves of a crystal of **1** during superelastic deformation at different temperatures: 25°C (black), 30°C (red), and 50°C (blue).

Supplementary references

[S1] Burke, J. J., Reed, N. L. & Weiss, V. *Surfaces and Interfaces II: Physical and Mechanical Properties,* (Syracuse University Press NY, 1968).

[S2] Langdon, T. G. An evaluation of the strain contributed by grain boundary sliding in superplasticity. *Mater. Sci. Eng.* **A174**, 225–230 (1994).

[S3] Langdon, T. G. Grain boundary sliding revisited: Developments in sliding over four decades. *J. Mater. Sci.* **41**, 597–609 (2006).

[S4] Kim, B.-N., Hiraga, K., Morita, K. & Sakka, Y. A high-strain-rate superplastic ceramic. *Nature* **413**, 288–291 (2001).

[S5] Higashi, K., Mabuchi, M. & Langdon, T. G. High-strain-rate superplasticity in metallic materials and the potential for ceramic materials. *ISIJ Int.* **36**, 1423–1438 (1996).
