# Supplementary material for: Superplasticity in an organic crystal
Source: Nat Commun. 2018 Sep 28;9:3984. doi: 10.1038/s41467-018-06431-7 (PMC6162311; doi:10.1038/s41467-018-06431-7)
Supplement: Supplementary file 3 — Description of Additional Supplementary Files [file 41467_2018_6431_MOESM3_ESM.pdf]

Description of Additional Supplementary Files

Supplementary Movie 1: Superplastic deformation of a crystal of 1 up to ca. 500% strain.

Supplementary Movie 2: Twinning deformation of a crystal of 1 in conjunction with multi-layer slipping.

Supplementary Movie 3: Superelastic behavior of a crystal of 1.

Supplementary Movie 4: Superplastic deformation of a crystal of 1 of which strain rate quickly shifted from 1.0 s-1 to 5.0 s-1.

Supplementary Movie 5: Superplastic deformation of a crystal of 1 of which strain rate quickly shifted from 2.0 s-1 to 20 s-1.

Supplementary Movie 6: Superelastic behavior of a crystal of 1 during a stress test.

Supplementary Movie 7: Superplastic deformation of a crystal of 1 up to ca. 400% strain.

Supplementary Movie 8: Superelastic behavior of a crystal of 1 after superplastic deformation up to ca. 400% strain.

Supplementary Movie 9: Coupling of superplasticity and superelasticity in a crystal of 1.
